# Seasonal Changes in Endotoxin Exposure and Its Relationship to Exhaled Nitric Oxide and Exhaled Breath Condensate pH Levels in Atopic and Healthy Children

**DOI:** 10.1371/journal.pone.0066785

**Published:** 2013-06-19

**Authors:** Gwo-Hwa Wan, Dah-Chin Yan, Tao-Hsin Tung, Chin-Sheng Tang, Chiu-Hsin Liu

**Affiliations:** 1 Department of Respiratory Therapy, College of Medicine, Chang Gung University, Tao-Yuan, Taiwan; 2 Division of Taipei Pediatrics, Department of Pediatrics, Chang Gung Children’s Hospital, Chang Gung Memorial Hospital, Taipei, Taiwan; 3 Department of Medicine, College of Medicine, Chang Gung University, Tao-Yuan, Taiwan; 4 Department of Medical Research and Education, Cheng-Hsin General Hospital, Taipei, Taiwan; 5 Department of Public Health, College of Medicine, Fu Jen Catholic University, New Taipei City, Taiwan; 6 Department of Respiratory Therapy, Chang Gung Memorial Hospital, Taipei, Taiwan; University of Cincinnati, United States of America

## Abstract

Endotoxin, a component of the cell walls of gram-negative bacteria, is a contaminant in organic dusts (house dust) and aerosols. In humans, small amounts of endotoxin may cause a local inflammatory response. Exhaled nitric oxide (eNO) levels, an inflammation indicator, are associated with the pH values of exhaled breath condensate (EBC). This study evaluated seasonal changes on indoor endotoxin concentrations in homes and the relationships between endotoxin exposure and eNO/EBC pH levels for healthy children and children with allergy-related respiratory diseases. In total, 34 children with allergy-related respiratory diseases and 24 healthy children were enrolled. Indoor air quality measurements and dust sample analysis for endotoxin were conducted once each season inside 58 surveyed homes. The eNO, EBC pH levels, and pulmonary function of the children were also determined. The highest endotoxin concentrations were on kitchen floors of homes of children with allergy-related respiratory diseases and healthy children, and on bedroom floors of homes of asthmatic children and healthy children. Seasonal changes existed in endotoxin concentrations in dust samples from homes of children with allergic rhinitis, with or without asthma, and in EBC pH values among healthy children and those with allergy-related respiratory diseases. Strong relationships existed between endotoxin exposure and EBC pH values in children with allergic rhinitis.

## Introduction

The prevalence of allergy-related respiratory diseases has gradually increased over the last two decades, especially among children who live in highly urbanized areas. Major risk factors for such diseases include modernization and considerable time spent indoors [1]. The development of allergy-related respiratory diseases involves the result of interactions between different genetic and environmental factors.

Endotoxin, a component of cell walls of gram-negative bacteria, is ubiquitous in indoor environments. In its pure form, endotoxin is a lipopolysaccharide (LPS). Endotoxin has strong pro-inflammatory properties that can induce airway inflammation and cytokine upregulation in humans [2]. Acute endotoxin exposure may induce blood and lung inflammatory responses that involve neutrophils and macrophages, which can result in fever, shaking chills, and severe asthma [3].

Responses to endotoxin inhalation typically differ among individuals based on genetic factors or degree of tolerance [4]. Studies have indicated that endotoxin exposure in infancy has a protective role against asthma development and allergen sensitization [5], [6]. However, endotoxin exposure during childhood and later in life likely has detrimental effects on both healthy individuals and those with asthma and respiratory diseases [7]–[9].

Endotoxin is in air and in house dust, and occurs as a contaminant of organic dusts and environment aerosols [10]. Inhalation exposure to endotoxin is common in occupational environments [11] and in homes [12], [13]. Household endotoxin exposure is a strong risk factor for asthma [8] and wheezing [14], [15]. Such factors as poverty, number of people in a household, pets, bedding materials, cleaning methods, frequency of cleaning, and geographic location influence indoor endotoxin exposure [12], [13], [16].

Endotoxin exposure from floor dust is more critical than that from airborne particles, as the breathing zone of children is close to the ground [17]. A clear correlation existed between endotoxin content in house dust from living room floors and airborne endotoxin concentrations [18]. Another investigation demonstrated that the highest and lowest endotoxin concentrations in homes were on kitchen floors and on bedding, respectively [8].

Measuring nitric oxide in exhaled breath is a non-invasive technique for assessing airway inflammation. Studies have shown that eNO may be a diagnostic factor for asthma [19] and for evaluating the anti-inflammatory effects of inhaled corticosteroids in asthmatic children [20]–[22]. Exhaled nitric oxide is also related to allergic sensitization in childhood asthma and allergic rhinitis [23] via the late-phase influx of eosinophils [24] and nitric oxide formation after aeroallergen exposure [25], [26]. Furthermore, eNO concentrations are negatively associated with the pH values of EBC in asthmatics [27]. However, no correlation existed between eNO and EBC pH in children [28], [29]. The EBC pH values in asthmatic children with or without inhaled corticosteroid treatment are clearly lower than those in healthy children [30], [31]. Environmental factors, particularly endotoxin exposure, have a significant effect on degree of airway inflammation [32], [33]. However, few studies have examined endotoxin’s effects on eNO levels and EBC pH values.

To date, no published data exists on seasonal variations of indoor endotoxin concentration distributions in homes and on the relationships between endotoxin exposure and eNO/EBC pH levels in children with allergy-related respiratory diseases and in healthy children. This study determined seasonal changes in indoor endotoxin concentrations (on living room floors, bedroom floors, mattresses, and kitchen floors) in homes of healthy children and those of children with allergy-related respiratory diseases in Taipei, Taiwan. Additionally, nitric oxide levels and pH values in the exhaled breath of healthy children and those with allergy-related respiratory diseases were used to evaluate possible associations with endotoxin exposure in the home.

## Materials and Methods

### Study Population

In total, 58 children were recruited: 15 asthmatic children; 9 with allergic rhinitis; 10 with asthma and allergic rhinitis; and 24 healthy children. Age range was 6–13 years. Children with allergy-related respiratory diseases were recruited from the Department of Pediatrics at Chang Gung Children’s Hospital, Taipei, Taiwan. All asthmatic children were diagnosed by a pediatrician according to the guidelines of the Global Initiative for Asthma (GINA) [34] and the modified National Asthma Education and Prevention Program (NAEPP) [35]. Although children with allergic rhinitis did not have hyper-reactive airway responses during methacholine challenge tests, they met the guidelines in Allergic Rhinitis and its Impact on Asthma (ARIA) [36]. Healthy children with no history of allergies or pulmonary disease were recruited from the Department of Pediatrics at Chang Gung Children’s Hospital and from the same elementary schools in Taipei City that were attended by recruited children with allergy-related respiratory diseases. Study protocol was approved by the Institutional Review Board of Chang Gung Hospital. Informed written consent was obtained from each child’s parents.

### Air Sampling

The timing of home visits for indoor air sampling was in the first two months of each season, and based on then time schedule of each family. The seasons were defined as follows: spring (February to April); summer (May to July); autumn (August to October); and winter (November to January). Environmental measurements of air temperature, relative humidity (RH), carbon dioxide (CO_2_), total volatile organic compounds (TVOCs), and suspended particulate matter were taken inside the homes of healthy children and of those with allergy-related respiratory diseases. Indoor temperature, RH, and CO_2_ concentration were determined using a digital psychrometer (TSI, Inc., Shoreview, MN, USA). The TVOCs levels were determined using a Model PGM 7240 ppbRAE® sampler (RAE Systems, Inc., San Jose, CA, USA). A portable DUSTcheck monitor (Model 1.108; Grimm Labortechnik Ltd., Ainring, Germany) was used to measure mass concentrations of airborne particulate matter.

Air samplers were placed in the center of each living room for 8 consecutive hours. Sampling height was 1.0–1.2 meters above the floor, a child’s breathing zone. The frequency of air sampling was once per season.

### House Dust Collection and Endotoxin Analysis

The timing of house dust sampling was the same as that of indoor air sampling. Dust samples were collected each season from four sites in homes: the top surface of a child’s mattress; the bedroom floor next to the mattress; living room floor; and kitchen floor. A vacuum cleaner fitted with a fresh glass-fiber filter was used to vacuum a 1 m^2^ surface area for 2 minutes at each sampling site. Collected dust samples were sieved through a 425-µm mesh screen to obtain fine dust.

All endotoxin-free glassware and metals were heat treated at 180°C for 4 hours. A fine dust sample (5 mg) was agitated with 1 mL triethylamine phosphate (TAP) buffer (pH 7.5) for 1 hour to extract endotoxins. Endotoxin activity in a dust sample was determined with a chromogenic *Limulus* amebocyte lysate assay (Associates of Cape Cod, East Falmouth, MA, USA). Standard response curves were generated using endotoxin standards in the range of 0.25–4.0 EU/mL (correlation coefficient, r = 0.99). A negative control with pyrogen-free water was used with each assay. No sample had an activity level below the assay’s detection limit.

### Exhaled Nitric Oxide, EBC pH, and Spirometric Measurements

The eNO/spirometric measurement and EBC collection were performed within 1 week after domestic environmental sampling. The eNO levels of all children were measured at an expiratory flow rate of 50 ml/s using a chemiluminescence analyzer (CLD 88 sp; ECO Physics, Dürnten, Switzerland) according to international standards [37]. A non-invasive cooling device (EcoScreen, Jaeger Toennies, Hoechberg, Germany) was used to collect the subjects’ EBCs. While sitting upright and wearing a nose clip, each subject breathed normally through a mouthpiece for 15 minutes for EBC sampling. Approximately 1–3 mL of EBCs were collected from each subject and then analyzed. All EBC samples were deaerated with argon at a flow rate of 350 ml/min to remove carbon dioxide. Then, EBC pH was determined using a digital pH meter (UB-5; Denver Instruments, Denver, CO, USA). A spirometer (MIR Spirolab II, Pinyork, Japan) was used to determine forced expiratory volume in 1 second (FEV_1_), forced vital capacity (FVC), and maximum mid-expiratory flow (MMEF). The frequency of eNO, EBC pH, and spirometric determinations for healthy children and children with allergy-related respiratory diseases was the same as that for indoor air sampling.

### Statistical Analysis

Statistical analyses used SPSS version 13.0 (SPSS, Inc., Chicago, IL, USA). Figures were graphed with GraphPad Prism 5.0 software (GraphPad Software, Inc., San Diego, CA, USA). The significance level for all tests was 0.05. The necessary study sample size was calculated by considering the eNO levels as the primary outcome [30]. In a Kruskal-Wallis test study, sample sizes of 15, 9, 10, and 24 were obtained from the asthma group, allergic rhinitis group, asthma+allergic rhinitis group, and control group whose means were compared. The total sample of 58 subjects had an 87% power to detect differences among means *versus* the alternative of equal means using an F test with a 0.05 significance level.

One-way analysis of variance (ANOVA) for normally distributed data was used to identify group difference in age. The Kruskal-Wallis test and Mann-Whitney U test for non-normally distributed data were used to identify group differences in continuous variables. A c*hi*-squared test was applied to identify group differences in categorical variables. Seasonal variations in indoor air indices and endotoxin concentrations in homes, and levels of eNO and EBC pH in children were assessed by non-parametric repeated-measures ANOVA for skew distributed data. The strength of correlation was assessed by the Spearman test for non-normally distributed data to determine the relationship between the endotoxin concentrations in dust samples and eNO/EBC pH levels of all recruited children.

## Results

Age range of recruited children was 6–13. Average age differed significantly between the four groups (*p*<0.01; Table 1). No significant differences existed for gender. Children with allergy-related respiratory diseases were mainly sensitized by house dust mite (*D. farinae* and *D. pteronyssinus*) allergens, dog and cat danders, the cockroach allergen, and *Candida albicans*. No significant differences in positive rates of blood allergen-specific IgE tests existed among the asthma group, allergic rhinitis group, and asthma+allergic rhinitis group. Mean pulmonary function parameters for children with allergy-related respiratory diseases were normal. The FEV_1_/FVC (% predicted) and MMEF (% predicted) differed significantly among the four groups of children (*p*<0.01). Mean severity of allergy-related respiratory diseases was mild-intermittent level. The highest and lowest median eNO levels were in asthmatic children (36.9 ppb) and healthy children (11.7 ppb), respectively. The eNO level of asthmatic children was highest (*p*<0.01). Additionally, the EBC pH value (6.3) of asthmatic children was significantly lower than for the other three groups (children with allergic rhinitis, EBC pH = 7.9; children with asthma and allergic rhinitis, EBC pH = 7.9; and healthy children, EBC pH = 7.3; *p*<0.01).

**Table 1:** Personal and domestic environmental characteristics of children.

Variables		Asthma (AS) group				Allergic rhinitis (AR) group			AS+AR group		Control group			*p* value	
Sample size, n		15				9			10		24				
Age, y/o		10.4	(0.9)¶,‡, *			8.2	(1.6)#		8.8	(1.9)§	11.6		(1.3)	<0.01	
Gender, M/F		12/3				4/5			6/4		10/14			0.110	
Positive rates of blood allergen-specific IgE test, n (%)															
Cockroach		2	(13.3%)			1	(5.3%)		2	(10.5%)	–			0.844	
Cat dander		2	(13.3%)			1	(5.3%)		0	(0%)	–			0.495	
* Candida albicans*		4	(26.7%)			0	(0%)		1	(5.3%)	–			0.179	
Dog dander		5	(33.3%)			2	(10.5%)		4	(21.1%)	–			0.706	
D. *farinae*		14	(93.3%)			9	(47.4%)		10	(52.6%)	–			0.521	
D. *pteronyssinus*		14	(93.3%)			9	(47.4%)		10	(52.6%)	–			0.521	
Pulmonary function, % predicted															
FEV_1_	86.0		(77.3–92.0)*			84.5	(76.0–89.0)#		81.5	(73.5–89.5)§	90.0	(85.0–94.0)		<0.01	
FEV_1_/FVC	92.0		(85.3–98.8)¶, *			98.5	(96.0–102.0)†		90.0	(86.3–94.8)§	99.0	(94.0–102.0)		<0.01	
MMEF	90.5		(81.3–100.8)‡			90.0	(78.0–102.0)		81.0	(70.3–89.8)§	93.0	(85.0–104.0)		<0.01	
Exhaled gas indices															
eNO, ppb	36.9			(17.0–56.0)¶,‡, *		18.4	(8.8–23.9)#		17.8	(7.5–30.6)§	11.7	(7.5–18.8)		<0.01	
EBC pH	6.3			(6.0–7.1)¶,‡, *		7.9	(7.6–8.1)#		7.9	(7.8–8.0)§	7.3	(6.4–7.8)		<0.01	
Environmental characteristics															
Temperature, °C	27.5				(23.6–29.4)	27.6		(23.7–29.8)	27.8	(23.2–30.4)	26.4	(22.9–29.5)			0.782
Humidity, %	69.3				(62.0–72.6)‡, *	65.0		(63.5–69.8)	65.1	(58.5–69.9)	66.2	(59.8–70.5)			0.050
CO_2_, ppm	583.4				(465.7–754.8)	517.8		(484.7–657.5)	542.9	(474.1–641.3)	533.8	(464.4–621.5)			0.680
TVOCs, ppb	143.2				(53.1–304.9)	124.0		(82.3–207.6)	125.3	(59.7–258.5)	118.3	(66.5–232.6)			0.739
PM_10_, µg/m^3^	33.7				(20.1–45.8)	29.2		(16.6–46.9)	28.4	(20.6–44.7)	32.4	(24.0–46.3)			0.676
PM_2.5_, µg/m^3^	21.5				(11.9–34.1)	21.3		(9.7–36.0)	20.4	(11.4–35.2)	21.7	(13.7–34.2)			0.863
PM_1_, µg/m^3^	17.6				(9.9–28.8)	17.7		(7.0–30.5)	17.6	(8.2–30.2)	16.8	(10.3–29.3)			0.948
Endotoxin, EU/mg dust	489.9				(215.3–756.2)*	430.2		(283.6–650.4)#	514.0	(366.7–969.3)	651.9	(393.8–1062.5)			0.021

Notes: data were represented as n (%), mean (SD) or median (25–75 percentiles); MMEF: maximum mid-expiratory flow; TVOCs: total volatile organic compounds;

¶ AS group vs. AR group;

‡ AS group vs. AS+AR group;

* AS group vs. control group;

† AR group vs. AS+AR group;

# AR group vs. control group;

§ AS+AR group vs. control group, *p*<0.05.

During the study period, median air temperatures were 26.4–27.8°C in all homes (Table 1). Median relative humidity was 65.0–69.3% and median CO_2_ concentrations were 517.8–583.4 ppm. The homes of children with asthma had the highest relative humidity (*p* = 0.05). No significant differences in concentrations of TVOCs and suspended particular matter (PM_10_, PM_2.5_, and PM_1_) in homes existed among the four groups. The median endotoxin level in the homes of both asthmatic children (489.9 EU/mg dust) and children with allergic rhinitis (430.2 EU/mg dust) was significantly lower than that in the homes of healthy children (651.9 EU/mg dust; *p* = 0.021).

**Figure 1:**
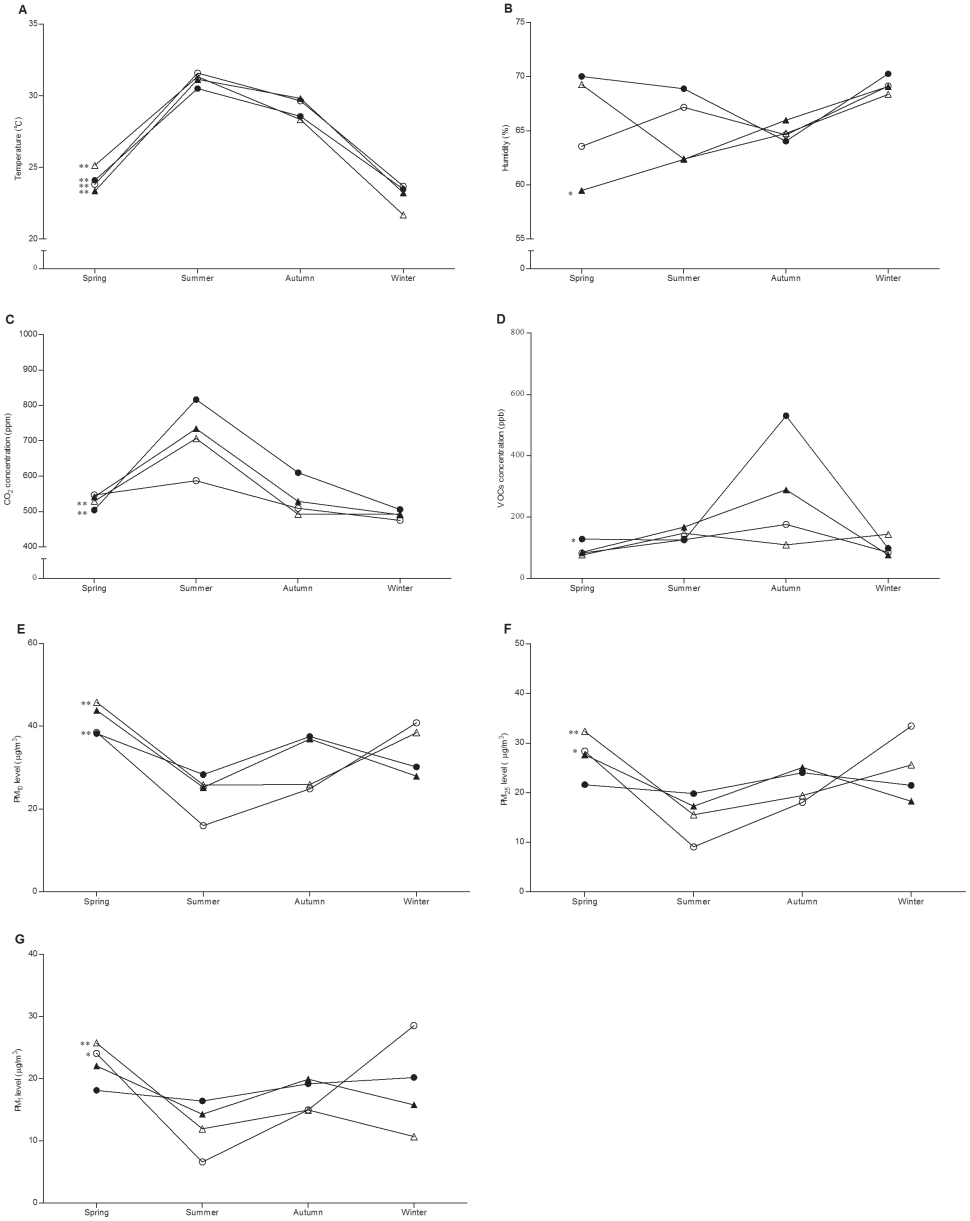
Seasonal variation of indoor air indices in children’s homes. *: *p*<0.05; **: *p*<0.01.

Groups were then evaluated separately to identify seasonal trends in indoor air indices such as temperature, humidity, CO_2_ concentrations, TVOCs concentrations, and PM levels. A seasonal trend for variations in air temperature was observed in the homes of asthmatic children, children with allergic rhinitis, children with asthma and allergic rhinitis, and healthy children (all *p*<0.01; Fig. 1A). Apart from seasonal changes in indoor humidity in the homes of children with asthma and allergic rhinitis (*p* = 0.011), the homes of other groups exhibited no seasonal changes of indoor humidity (Fig. 1B). The seasonal variation in CO_2_ concentrations was significant in homes of the asthma group (*p*<0.01) and the control group (healthy children) (*p*<0.01; Fig. 1C). Moreover, a seasonal trend for variations in TVOCs concentrations only existed in the homes of children with asthma (*p* = 0.026; Fig. 1D). Seasonal variation in the PM_10_ level was obvious in the homes of both children with allergic rhinitis (*p*<0.01) and healthy children (*p*<0.01; Fig. 1E). The levels of PM_2.5_ and PM_1_ had significant seasonal changes in the homes of both the allergic rhinitis group (PM_2.5_: *p* = 0.016, Fig. 1F; PM_1_: *p* = 0.017, Fig. 1G) and the control group (PM_2.5_: *p*<0.01, Fig. 1F; PM_1_: *p*<0.01, Fig. 1G).

**Figure 2:**
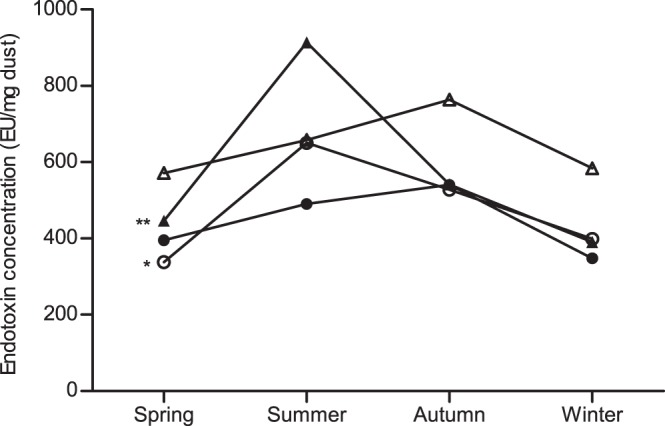
Seasonal variation of endotoxin concentrations in dust samples of children’s homes. •: asthma (AS) group; ○: allergic rhinitis (AR) group; ▴: AS+AR group; ▵: control group. *: *p*<0.05; **: *p*<0.01.

A seasonal trend for variations in endotoxin concentrations only existed in the homes of both children with allergic rhinitis (337.6 EU/mg dust in spring; 650.4 EU/mg dust in summer; 527.8 EU/mg dust in autumn; and 399.0 EU/mg dust in winter; *p* = 0.015) and children with asthma and allergic rhinitis (446.0 EU/mg dust in spring; 913.5 EU/mg dust in summer; 541.0 EU/mg dust in autumn; and 390.2 EU/mg dust in winter; *p*<0.01; Fig. 2).

**Figure 3:**
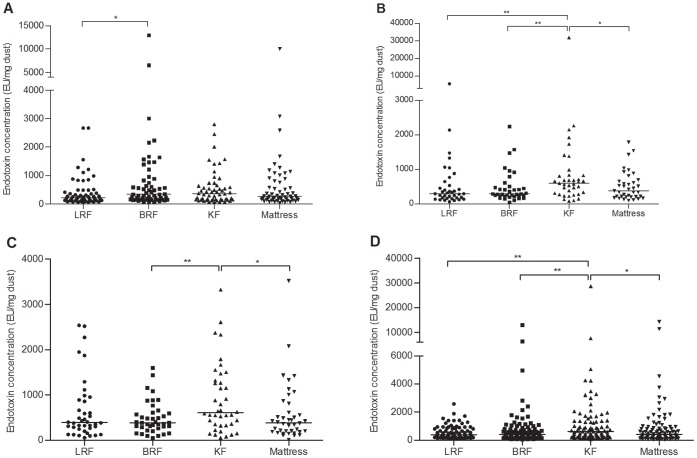
Endotoxin concentrations in dust samples of different locations of children’s homes. (A) asthma group; (B) allergic rhinitis group; (C) AS+AR group; (D) control group. LRF: living room floor; BRF: bedroom floor; KF: kitchen floor. –: median. *: *p*<0.05; **: *p*<0.01.

In the homes of asthmatic children, median endotoxin concentration on bedroom floors (341.8 EU/mg dust) was clearly higher than that on living room floors (219.6 EU/mg dust; *p* = 0.046; Fig. 3A). In the homes of children with allergic rhinitis, median endotoxin concentration on kitchen floors (602.8 EU/mg dust) was significantly higher than that on living room floors (292.5 EU/mg dust; *p*<0.01), on bedroom floors (296.1 EU/mg dust; *p*<0.01), and on mattresses (376.0 EU/mg dust; *p* = 0.011; Fig. 3B). In the homes of children with asthma and allergic rhinitis, median endotoxin concentration was highest on kitchen floors (607.9 EU/mg dust) and lowest on bedroom floors (388.7 EU/mg dust; *p*<0.01) and on mattresses (388.0 EU/mg dust; *p* = 0.02; Fig. 3C). The distributions of endotoxin concentrations (living room floors, 379.4 EU/mg dust; bedroom floors, 395.3 EU/mg dust; kitchen floors, 584.2 EU/mg dust; mattresses, 381.3 EU/mg dust) in the homes of healthy children were the same as those in the homes of children with allergic rhinitis (Fig. 3D).

**Figure 4:**
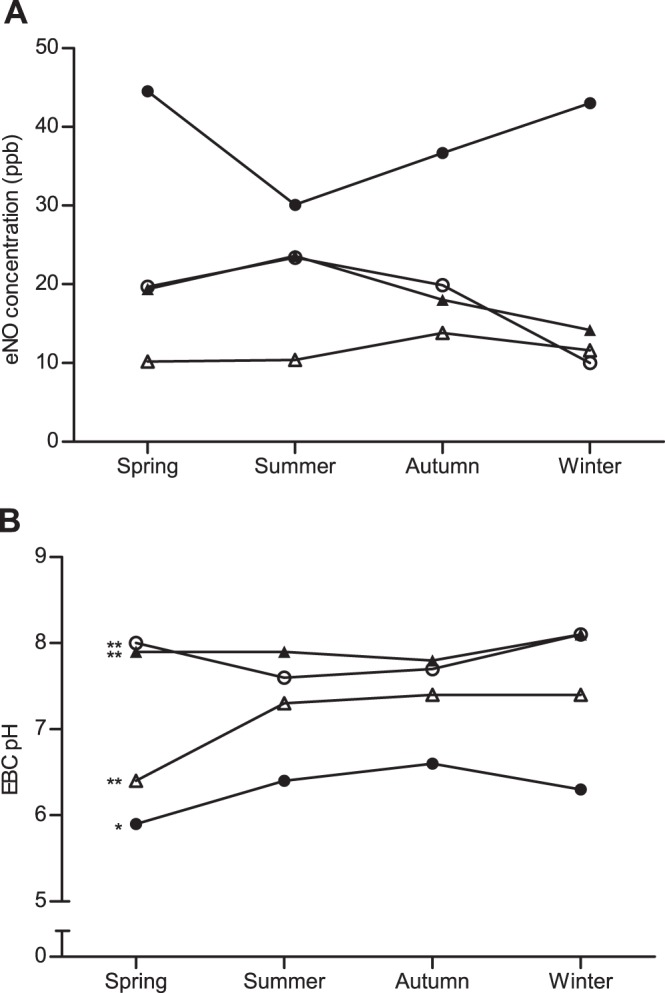
Seasonal variations of exhaled nitric oxide levels (A) and EBC pH values (B) in children. •: asthma group; ○: allergic rhinitis group; ▴: AS+AR group; ▵: control group. *: *p*<0.05; **: *p*<0.01.

Exhaled nitric oxide levels of >20 ppb were found in 86.7% of asthmatic children, 41.7% of children with allergic rhinitis, 45.0% of children with asthma and allergic rhinitis, and 22.9% of healthy children (data not shown). In terms of possible seasonal variations in eNO levels, no significant trends existed for children with allergy-related respiratory diseases and for healthy children (Fig. 4A). Notably, 58.3% of asthmatic children and 26.0% of healthy children had EBC samples with pH values <6.5 (data not shown). The four groups had significant seasonal variations in EBC pH values. For asthmatic children (5.9 in spring; 6.4 in summer; 6.6 in autumn; and 6.3 in winter; *p* = 0.025) (Fig. 4B); for children with allergic rhinitis (8.0 in spring; 7.6 in summer; 7.7 in autumn; and 8.1 in winter; *p*<0.01); for children with asthma and allergic rhinitis (7.9 in spring; 7.9 in summer; 7.8 in autumn; and 8.1 in winter; *p*<0.01); and for healthy children (6.4 in spring; 7.3 in summer; 7.4 in autumn; and 7.4 in winter; *p*<0.01).

**Figure 5:**
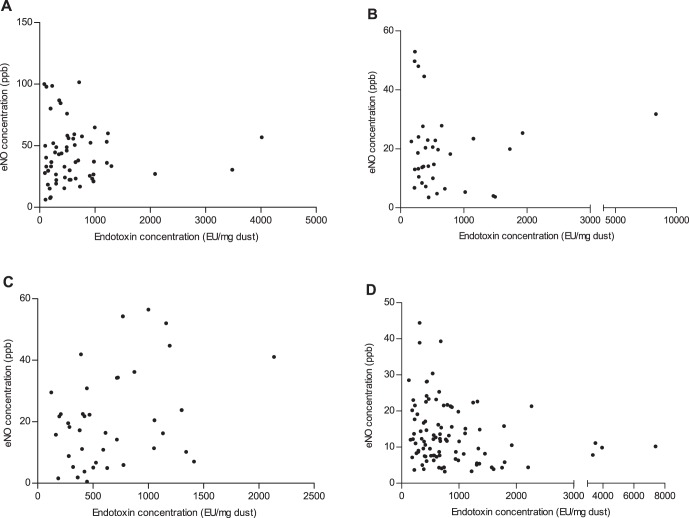
Associations between endotoxin concentrations and exhaled nitric oxide levels in children. (A) asthma group; (B) allergic rhinitis group; (C) AS+AR group; (D) control group.

**Figure 6:**
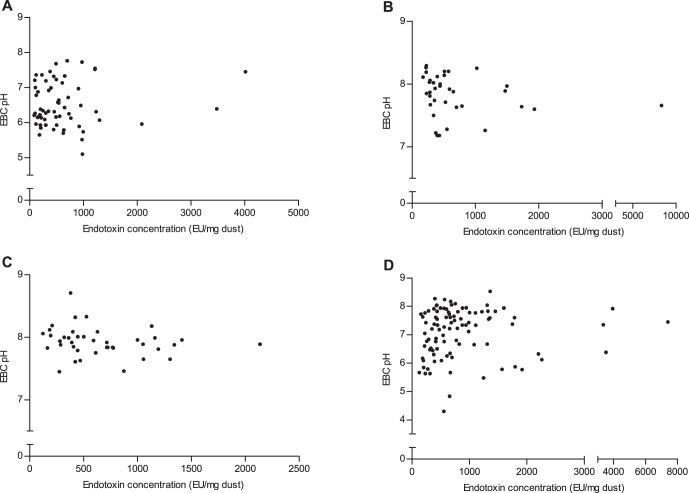
Associations between endotoxin concentrations and EBC pH values in children. (A) asthma group; (B) allergic rhinitis group; (C) AS+AR group; (D) control group.

No strong correlations existed between endotoxin exposure and eNO levels for asthmatic children (r = 0.085, *p* = 0.518; Fig. 5A), children with allergic rhinitis (r = –0.159, *p* = 0.353; Fig. 5B), or children with asthma and allergic rhinitis (r = 0.258, *p* = 0.107; Fig. 5C), a negative correlation was found between endotoxin exposure and eNO levels for healthy children (r = –0.232, *p* = 0.023; Fig. 5D). Further, no strong correlations existed between endotoxin exposure and EBC pH values for asthmatic children (r = 0.033, *p* = 0.800; Fig. 6A), children with asthma and allergic rhinitis (r = –0.274, *p* = 0.087; Fig. 6C), and healthy children (r = 0.172, *p* = 0.094; Fig. 6D). The EBC pH values were negatively correlated with endotoxin exposure for children with allergic rhinitis (r = –0.332, *p* = 0.048; Fig. 6B).

## Discussion

Based on long-term air monitoring results, 33.6% of the samples had CO_2_ levels >600 ppm, a threshold set by the Taiwan’s Environmental Protection Agency (EPA). These high CO_2_ levels were found in homes of healthy children (30.2%) and in homes of those with allergy-related respiratory diseases (41.7% of asthmatic children, 27.8% of children with allergic rhinitis, and 35.0% of children with asthma and allergic rhinitis). This finding demonstrates that significant percentages of homes of healthy children and of those with allergy-related respiratory diseases lacked adequate ventilation. Only 6.7% of the TVOCs samples from homes of asthmatic children exceeded the indoor air quality (IAQ) guideline (3 ppm of TVOCs) set by Taiwan’s EPA. All PM_10_ samples were under Taiwan’s EPA IAQ guidelines for 24-hour mean level (150 µg/m^3^). Only 0.4% of the PM_2.5_ samples from homes of healthy children exceeded the IAQ guideline (100 µg/m^3^ of PM_2.5_) set by Taiwan’s EPA. In this study, the concentrations of suspended particulate matter were determined only during an 8-hour period. Thus, future studies may extend sampling duration in homes to evaluate variations in suspended particulate matter.

In this study, the highest concentrations of house dust endotoxin were on kitchen floors in the homes of children with allergy-related respiratory diseases and of healthy children. These analytical results are similar to those in previous studies [8], [12], [38]. Endotoxin concentrations on kitchen floors in this study (353.5–607.9 EU/mg dust) were markedly higher than those in US studies (80.5–105 EU/mg dust) [8], [38]. A likely reason is the different climatic conditions of the two countries [7], [16]. In addition to kitchen floors, the bedroom floors (296.1–395.3 EU/mg dust) and the surfaces of mattresses (260.4–388.0 EU/mg dust) had higher endotoxin concentrations. Educating families of children, especially those of children with allergy-related respiratory diseases, to frequently clean bedroom floors and their bedding is essential.

Peak endotoxin concentrations in dust samples were in summer/autumn and the lowest were found in spring/winter in the homes of both children with allergy-related respiratory diseases and healthy children. This shows that the hottest season (May to October) promoted growth of gram-negative bacteria and increased endotoxin concentrations. Thus, children’s families should increase their housekeeping practices during the hottest season to decrease bacterial growth and endotoxin concentrations in their homes. Notably, endotoxin concentrations in the homes of children with allergy-related respiratory diseases, particularly asthmatic children and children with allergic rhinitis, were clearly lower than those in the homes of healthy children, demonstrating that environmental control education for children with allergy-related respiratory diseases and their families in clinical health teaching practice was beneficial for decreasing endotoxin concentrations in homes.

In this study, approximately 62.5% of children with allergy-related respiratory diseases and 22.9% of healthy children had high levels of eNO (>20 ppb) and about 25.9% of these children (25.7% in children with allergy-related respiratory diseases and 26.0% of healthy children) had low EBC pH levels (<6.5). This analytical finding reveals that significant percentages of these children had airway inflammation problems. Specially, asthmatic children had higher eNO levels and lower EBC pH values compared to those of children with allergic rhinitis, children with asthma and allergic rhinitis, and healthy children. Further, patterns of seasonal variations in EBC pH values were clearly different in asthmatic children for those of the other three groups in this study. However, eNO levels and EBC pH values were not directly related to endotoxin exposure in asthmatic children. This analytical result may be confounded by exposure to other air pollutants [39]–[41] and environmental allergens [42] in the homes of asthmatic children.

Endotoxin exposure was associated with decreased eNO levels among healthy children. Whether the eNO level was affected by endotoxin exposure in healthy children was not fully elucidated. Thus, the mechanism of endotoxin’s effect on eNO level in healthy children warrants further clarification. Moreover, the EBC pH values were negatively related to endotoxin exposure for children with allergic rhinitis. Future studies can identify the endotoxin dose needed to affect EBC pH values of children with allergic rhinitis. Additionally, this study did not measure the concentration distributions of environmental allergens in the homes of healthy children and children with allergy-related respiratory diseases. Therefore, future studies should evaluate the combined effect of endotoxin and environmental allergens on airway inflammation in humans. The interactions between exhaled breath indices (eNO and EBC pH), environmental factors, and medication use in different seasons also warrant further investigation. This study is limited by its laboratory method used for endotoxin determination. The limulus amebocyte lysate method can only detect intact LPS, whereas a newer cytokine induction assay using a monocytic cell line can detect both intact and small LPSs (<5 kDa), as well as peptidoglycans and short bacteria DNA fragments [43].

In conclusion, the highest endotoxin concentrations were on the kitchen floors of homes of healthy children and children with allergy-related respiratory diseases. Obvious seasonal changes existed in the endotoxin concentrations in dust samples from homes of allergic rhinitis group and asthma+allergic rhinitis group and in EBC pH values of healthy children and children with allergy-related respiratory diseases. Strong correlations existed between endotoxin exposure and eNO/EBC pH for healthy children and children with allergic rhinitis, respectively.
